# GATA transcription factors in testicular adrenal rest tumours

**DOI:** 10.1530/EC-17-0215

**Published:** 2017-10-16

**Authors:** Manon Engels, Paul N Span, Rod T Mitchell, Joop J T M Heuvel, Monica A Marijnissen-van Zanten, Antonius E van Herwaarden, Christina A Hulsbergen-van de Kaa, Egbert Oosterwijk, Nike M Stikkelbroeck, Lee B Smith, Fred C G J Sweep, Hedi L Claahsen-van der Grinten

**Affiliations:** 1 Department of PaediatricsRadboud Amalia Children’s Hospital, Radboud university medical center, Nijmegen, The Netherlands; 2 Laboratory MedicineRadboud Institute for Molecular Life Sciences (RIMLS), Radboud university medical center, Nijmegen, The Netherlands; 3 Radiation OncologyRadiotherapy and OncoImmunology Laboratory, RIMLS, Radboud university medical center, Nijmegen, The Netherlands; 4 MRC Centre for Reproductive HealthUniversity of Edinburgh, The Queen’s Medical Research Institute, Edinburgh, UK; 5 Department of PathologyRadboud university medical center, Nijmegen, The Netherlands; 6 Department of UrologyRadboud university medical center, Nijmegen, The Netherlands; 7 Department of Internal MedicineRadboud university medical center, Nijmegen, The Netherlands

**Keywords:** congenital adrenal hyperplasia, testicular adrenal rest tumour, Leydig cell tumour, GATA transcription factors

## Abstract

Testicular adrenal rest tumours (TARTs) are benign adrenal-like testicular tumours that frequently occur in male patients with congenital adrenal hyperplasia. Recently, GATA transcription factors have been linked to the development of TARTs in mice. The aim of our study was to determine GATA expression in human TARTs and other steroidogenic tissues. We determined GATA expression in TARTs (*n* = 16), Leydig cell tumours (LCTs; *n* = 7), adrenal (foetal (*n* = 6) + adult (*n* = 10)) and testis (foetal (*n* = 13) + adult (*n* = 8)). We found testis-like *GATA4*, and adrenal-like *GATA3* and *GATA6* gene expressions by qPCR in human TARTs, indicating mixed testicular and adrenal characteristics of TARTs. Currently, no marker is available to discriminate TARTs from LCTs, leading to misdiagnosis and incorrect treatment. *GATA3* and *GATA6* mRNAs exhibited excellent discriminative power (area under the curve of 0.908 and 0.816, respectively), while immunohistochemistry did not. GATA genes contain several CREB-binding sites and incubation with 0.1 mM dibutyryl cAMP for 4 h stimulated *GATA3*, *GATA4* and *GATA6* expressions in a human foetal testis cell line (hs181.tes). Incubation of adrenocortical cells (H295RA) with ACTH, however, did not induce *GATA* expression *in vitro*. Although ACTH did not dysregulate *GATA* expression in the only human ACTH-sensitive *in vitro* model available, our results do suggest that aberrant expression of GATA transcription factors in human TARTs might be involved in TART formation.

## Introduction

Congenital adrenal hyperplasia (CAH) is a genetic disorder in which adrenocortical steroid synthesis is impaired due to a deficiency in particular steroidogenic enzymes, most often steroid 21-hydroxylase (CYP21A2). A wide range of the male CAH patients from 12.5% up to 94% are reported to develop testicular adrenal rest tumours (TARTs), which are an important cause of infertility (1, 2). TARTs are benign tumours with steroidogenic characteristics, located near the mediastinum testis (1, 3). Until now, the aetiology and origin of TARTs have remained uncertain. TARTs were originally thought to arise from adrenal rest cells, based on the presence of adrenal characteristics, such as expression of adrenal enzymes and receptors (4, 5). However, recently we also described testicular characteristics of TARTs (6). This has shifted the hypothesis toward a more pluripotent steroidogenic cell type as the origin of TARTs (6), possibly from cells originating in the urogenital ridge or adrenogonadal primordium.

Besides exhibiting both adrenal and testicular characteristics, TARTs also share morphological similarities with steroid-producing testicular Leydig cells. As a consequence, it is difficult to discriminate TARTs from Leydig cell tumours (LCTs). Both TARTs and LCTs are rare tumours (7). Although rare entities, several cases of LCTs in CAH patients have been described (8, 9, 10, 11, 12, 13). Discrimination between LCTs and TARTs is important as these require different treatment strategies. TARTs are detected using ultrasound or MRI investigation. Currently, TARTs will only be surgically removed from the testis when pain complaints are present (3), while LCTs will be surgically removed using a testis-sparing procedure or total orchiectomy (7). No single marker is available yet to accurately discriminate TARTs from LCTs, increasing the chance of misdiagnosis and consequently incorrect treatment, of which at least 2 cases have been reported in literature (14, 15).

GATA transcription factors are involved in development (by regulating cell fate specification) and differentiation in all eukaryotic organisms. These factors are able to bind to a consensus DNA element, WGATAR, known as the GATA motif (16, 17). Historically, GATA transcription factors are divided into two families: GATA1, GATA2 and GATA3 are classified as haematopoietic factors, while GATA4, GATA5 and GATA6 are classified as endodermal factors. Their expression is also described in almost all foetal and adult tissues, and they are involved in adrenogonadal development. Three GATA factors (GATA 1, 4, 6) are expressed in the somatic cell population of the testis, while GATA3 is expressed in the adrenal medulla (reviewed in Viger and coworkers 18).

A possible relation between the expression of GATA transcription factors during adrenogonadal development and TART development was proposed in commentaries in the study of Padua and coworkers (19) by Heikinheimo and coworkers (20) and Pihlajoki (21). Padua and coworkers (19) developed a mouse model lacking both GATA4 and GATA6 expressions in steroidogenic cells. These mice suffer from adrenal aplasia, and female mice die within days after birth. However, male mice survive because of corticoid production by adrenal-like cells in the testes, which Heikinheimo (20) and Pihlajoki (21) proposed might be similar to TART cells. Interestingly, GATA genes contain cAMP response element-binding protein (CREB) sites, and cAMP induces expressions of GATA4 and GATA6 in gonadal cell lines (22, 23, 24). Levels of ACTH, the receptor of which signals via cAMP, are raised in CAH patients, due to lack of negative feedback on the Hypothalamic–Pituitary–Adrenal axis, caused by low or absent cortisol levels due to the adrenal enzyme deficiency. Furthermore, ACTH levels are associated with the occurrence of TARTs (25, 26, 27). Therefore, we hypothesized that dysregulation of GATA transcription factors by increased ACTH levels *in utero* might be involved in the aetiology of TARTs.

The aim of our study was therefore to determine the expression of GATA transcription factors in TARTs and other steroidogenic tissues. We determined their discriminative potential to discern TARTs from LCTs, and studied the role of cAMP and ACTH in the aetiology of TARTs *in vitro*.

## Methods

### GATA expression analysis in human material

#### Tissues and patients

Sixteen TART samples from 8 adult CAH patients (tumour left and right testis) were previously collected as described by Claahsen and coworkers (3, 5) (informed consent was obtained). Paraffin-embedded material for immunohistochemistry was available for all tumours, while frozen material for RNA isolation was available for 12 samples. Additionally, 2 frozen histologically proven TART samples from one anonymous CAH patient were obtained. Frozen material of normal testis (*n* = 8), normal adrenal (*n* = 10) and benign LCT (*n* = 7) was obtained. Furthermore, paraffin-embedded material was available for 4 benign LCTs and 3 metastases of malignant LCTs. These coded (identifiable anonymous) testis tissues, adrenal tissues, benign LCTs, metastases of malignant LCTs and TART samples were obtained from the Pathology and Urology departments and used in accordance with the Code of Conduct of the Federation of Medical Scientific Societies in the Netherlands (http://www.federa.org/codes-conduct; research approved by institutional review board: CMO Radboudumc #2016-2977 and CMO-nr 2004/007).

To study the aetiology of TARTs, we also included foetal adrenal and testis tissues. Six human foetal adrenals (first and second trimesters) and cDNA from 13 foetal testis tissues (second trimester) were obtained from the MRC Centre for Reproductive Health, University of Edinburgh. Tissues were obtained following elective termination of pregnancy and anonymized. Women gave informed consent in accordance with national guidelines (42), and ethical approval was obtained from the Lothian Research Ethics Committee.

#### RNA isolation

Frozen tissue sections (at least 10 × 20 µm) or cultured cells were used for RNA isolation (Total RNA Purification kit, Norgen, Thorold, Canada) according to manufacturer’s instructions. Samples were treated with DNase (RNase-free DNase set, Qiagen). RNA concentrations and purity were determined using a NanoDrop 2000 Spectrophotometer.

#### Reverse transcription and qPCR

0.1 µg (foetal testis), 0.2 µg (first-trimester adrenal) or 0.5 µg (second trimester adrenal and adult samples) of total RNA was used for cDNA synthesis using Superscript II reverse transcriptase (Thermo Fisher Scientific), performed according to the manufacturer’s protocol with a 2720 Thermal cycler (Applied Biosystems) in a final volume of 20 µL. Gene-specific primers of GATA1, GATA3, GATA4 and GATA6 were self-designed (Supplementary Table 1, see section on supplementary data given at the end of this article). For qPCR, the cDNA of adult samples was diluted 5 times, while the cDNA of foetal samples was diluted 20 times, and 2 µL was added to 7.5 µL IQSYBR Green Supermix (Bio-Rad Laboratories), in a total amount of 15 µL on a CFX96 Touch Real-Time PCR Detection System (Bio-Rad Laboratories). As foetal testis cDNA was not DNase-treated, a non-RT control was used to determine genomic DNA contamination.

#### Immunohistochemistry

GATA3 immunostaining was performed using a standardized protocol optimized for the localization of GATA3 in urothelial carcinoma (antibody L50-823, 1:50 dilution, Biocare Medical/Klinipath, Duiven, The Netherlands). Kidney sections were used as positive control. GATA6 immunostaining (sc-9055; 1:200 dilution, SantaCruz Biotechnology) was performed manually including negative control sections with only primary antibody diluent. All sections were visualized with VisionTek (Sakura, Tokyo, Japan).

### Regulation of GATA transcription factors

#### Cell culture

The Hs181.tes cell line was obtained from American Type Culture Collection (ATCC CRL-7131), while the H295RA cell line was obtained from the University of Michigan (28). Cells were grown as a monolayer culture, although the H295RA cells tend to grow in clumps. Media for Hs181.tes cells consisted of DMEM with 4.5 g/L glucose with l-glutamine (Lonza; Leusden, Netherlands), whilst DMEM/F12 (Lonza) was used for H295RA cells. Both media were supplemented with 10% foetal bovine serum (Gibco; Thermo Fisher Scientific) and 1% antibiotics (penicillin-streptomycin 10,000 U/mL; Gibco). Cells were cultured at 37°C in a humidified 95% air/5% CO_2_ atmosphere. Medium was changed 2–3 times a week and Hs181.tes cells were passaged when confluent using 0.25% trypsin (BD Diagnostic Systems, Breda, The Netherlands), while for H295RA cells, 0.05% trypsin-EDTA (Gibco) was used.

#### Dibutyryl cAMP and ACTH studies

Hs181.tes and H295RA cells were washed, harvested and plated (1:6 dilution) into a 6-well plate (Costar, Corning Life Sciences). After 24 h, cells were starved overnight using serum-free medium (Hs181.tes) or low-serum experimental medium (1% FBS; H295RA). RNA was isolated after cells were treated with 0.1 mM dibutyryl cAMP (dbcAMP; Sigma) for either 30 min, 4 h or not treated at all. ACTH incubation experiments were only performed in the H295RA cells, as Hs181.tes cells are insensitive to ACTH. RNA was isolated after cells were not treated or treated with 2 or 10 nM ACTH (Synacthen, Radboudumc Pharmacy, Nijmegen, The Netherlands) for either 30 min, 4 h or 24 h.

### Data analysis

#### Gene expression

mRNA expression of all genes was calculated using the delta Ct method (2^−∆Ct^). All values were normalized to the corresponding *HPRT* value (29). Data were transferred to GraphPad Prism 5 and IBM SPSS 22.0 (SPSS Inc.) for further analyses. Differences between different tissues and conditions were tested for statistical significance with non-parametric tests. To determine the diagnostic properties of GATA in discriminating TARTs from LCTs, Mann–Whitney *U* was performed and following Receiver Operating Characteristic (ROC) analyses were performed. The area under the curve (AUC) represents the probability that the outcome correctly classifies the tissue as TART or benign LCT (range 0.5 (no accuracy) to 1 (prefect accuracy)). To determine the role of GATA in the aetiology of TARTs, a comparison between TARTs and testes (foetal and adult), TARTs and adrenals (foetal and adult) was made. Furthermore, expressions in foetal tissues were compared with each other (adrenal vs testis), as well as expressions in adult tissues (adrenal vs testis). Also, expressions in foetal and adult testis, and foetal and adult adrenal tissues were compared. These comparisons were analysed with the Kruskal–Wallis test followed by Dunn’s *post hoc* test. Gene expression analyses within cell culture studies were also compared using the Kruskal–Wallis test followed by Dunn’s *post hoc* test. Values of *P* ≤ .05 (*) or *P* ≤ .01 (**) or *P* ≤ .001 (***) were considered statistically significant.

#### Protein immunohistochemistry

IHC stainings for GATA3 and GATA6 in TARTs and LCTs were visually examined by two independent investigators (MAMvZ, ME). GATA3 and GATA6 were scored based on pseudoquantative histoscore: intensity of staining was recorded as negative, weak, moderate or strong. Furthermore, an estimation of the percentage of positive cells was made. For GATA3, only nuclear staining was scored as GATA3 staining is already validated for urothelial cell carcinoma, indicating only nuclear staining as a positive reaction. In contrast, as GATA6 staining is still experimental, we considered both nuclear and cytoplasmic stainings as positive. For GATA3, nuclear reactivity of the tubular cells of the kidney was used as external positive control for the staining procedure. For GATA6, tissue sections without primary antibody were used as a negative control for the staining procedure. Cytoplasmic staining in GATA6 was corrected for any lipofuscin present by comparing the negative control directly with the GATA6 staining. In the cases of staining heterogeneity, the highest level was reported. Staining scores were compared between the two investigators (MAMvZ, ME) and both agreed on reported (consensus) scores.

## Results

### Adrenal and testicular expression levels of GATA transcription factors in TARTs

**Figure 1:**
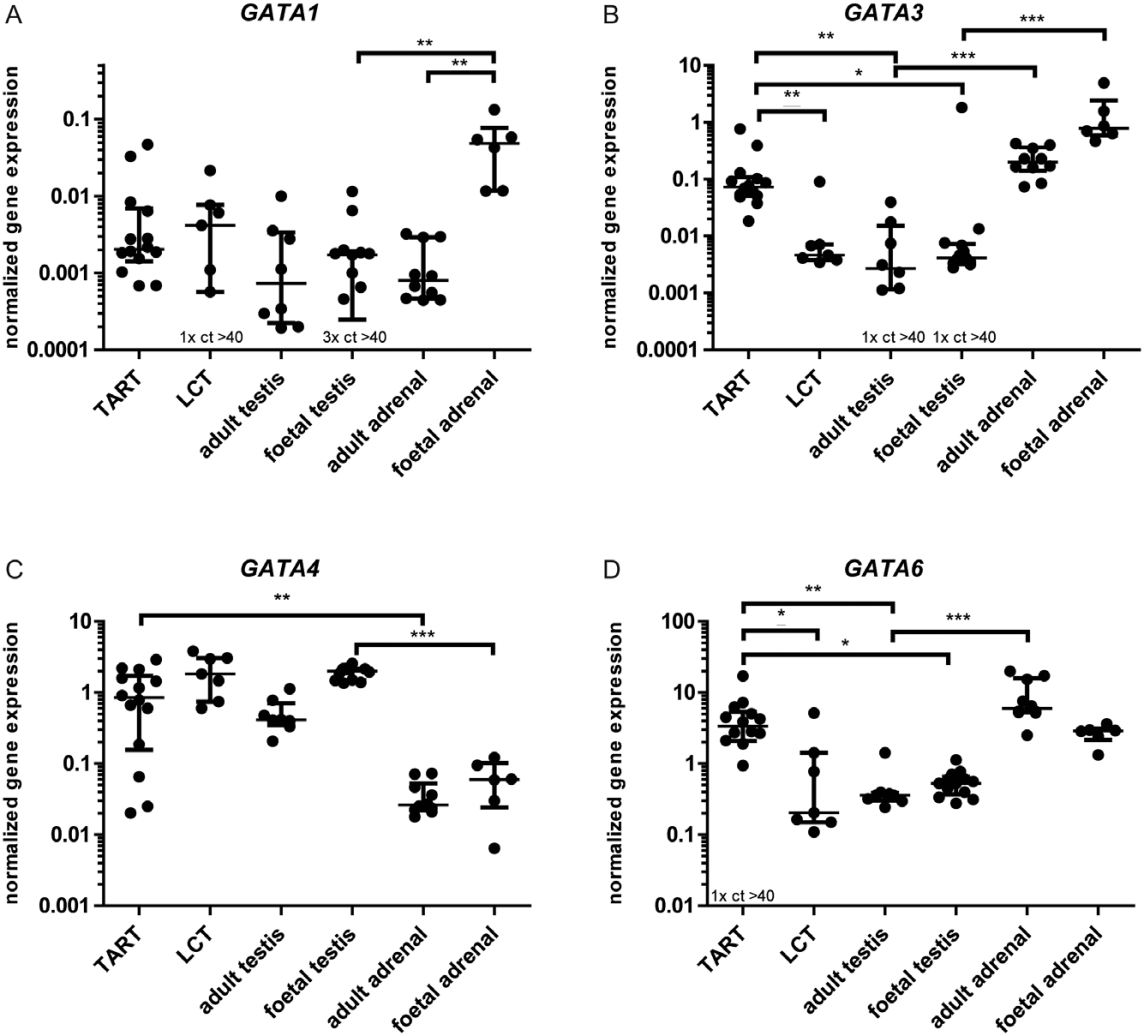
Gene expressions of *GATA1* (A), *GATA3* (B), *GATA4* (C) and *GATA6* (D) in human TARTs, adult testes, foetal testes, adult adrenals and foetal adrenals. mRNA expression was calculated using the delta Ct method. All values were normalized to corresponding *HPRT* expression. The symbols in the graph represent all samples used and the error bars indicate median and 25th and 75th percentiles. Significance was tested with the Mann–Whitney-*U* (MWU) test or Kruskal–Wallis and Dunn’s *post hoc* test comparing selected pairs of variables. Underlined significances are from the biomarker analysis comparing TARTs and LCTs (MWU). Non-underlined significances are from the aetiology analysis comparing TART, adult adrenal, adult testis, foetal adrenal and foetal testis tissues. *P ≤ 0.05; **P ≤ 0.01; ***P ≤ 0.001; ct>40 means that mRNA was not detected in these samples. LCTs, Leydig cell tumours; TARTs, testicular adrenal rest tumours.

To test whether expression of *GATA1*, *GATA3*, *GATA4* and *GATA6* is a marker of different tissues or disease states, qPCR was performed (Fig. 1). Separate analyses were performed to determine their discriminative potential between TARTs and LCTs (Fig. 1 underlined significance) and to determine their role in the aetiology of TARTs by comparison with foetal and adult testis and adrenal tissues (Fig. 1 non-underlined significance).

Expression of *GATA1* was significantly higher in foetal adrenals compared to foetal testes (28.4-fold, *P* ≤ 0.01) and adult adrenals (61.1-fold, *P* ≤ 0.01) (Fig. 1A).

*GATA3* expression was maximal in foetal adrenals, which was 190-fold higher compared to foetal testis tissues (*P* ≤ 0.001). *GATA3* expression was also significantly higher in adult adrenals compared to adult testes (73.7-fold, *P* ≤ 0.001), and expression in TARTs was significantly higher compared to foetal (17.8-fold, *P* ≤ 0.05) and adult (26.9-fold, *P* ≤ 0.01) testes (Fig. 1B).

Gene expression of *GATA4* was significantly higher (32.4-fold) in TARTs compared to adult adrenals (*P* ≤ 0.01). Significantly higher *GATA4* expression (33.5-fold) was found in foetal testis tissues compared to foetal adrenal tissues (*P* ≤ 0.001) (Fig. 1C).

*GATA6* expression levels were 16.6-fold higher in adult adrenals compared to adult testes (*P* ≤ 0.001), while *GATA6* expression in TARTs was higher compared to foetal (6.3-fold, *P* ≤ 0.05) and adult (9.3-fold, *P* ≤ 0.05) testes (Fig. 1D).

**Figure 2:**
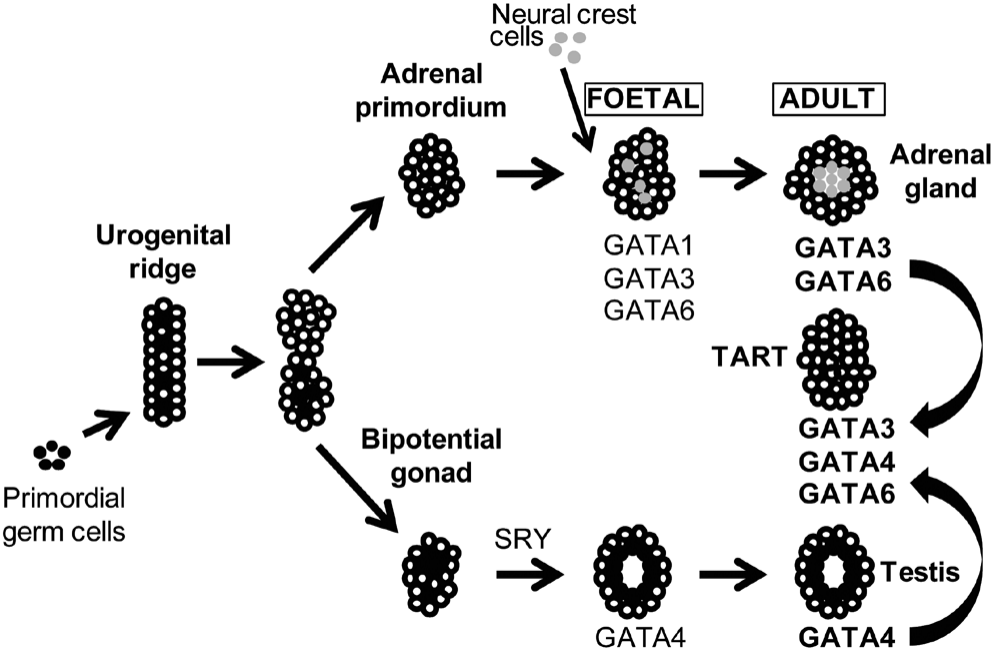
Gene expression of GATA transcription factors during gonadal and adrenal developments. The figure summarizes the results of our gene expression analysis in relation to adrenogonadal development. Cells from the adrenal primordium combined with neural crest cells give rise to the foetal adrenal, which will mature into the adult adrenal. The testis develops from the bipotential gonad. In this study, we measured gene expression levels of *GATA1*, *GATA3*, *GATA4* and *GATA6* in human TART, foetal and adult adrenal, and foetal and adult testis tissues. Of note, we are uncertain of cell-specific expression as we measured expression in total tissue. *GATA3* and *GATA6* were expressed in both foetal and adult adrenals, while *GATA4* was expressed in the foetal as well as the adult testes. *GATA3*, *GATA4* and *GATA6* gene expressions were all found in TARTs. TARTs, testicular adrenal rest tumours. Reproduced from Viger RS, Guittot SM, Anttonen M, Wilson DB, Heikinheimo M, Role of the GATA family of transcription factors in endocrine development, function, and disease, *Molecular Endocrinology*, 2008, volume 22, issue 4, pages 781–798, by permission of Oxford University Press. Copyright 2008, The Endocrine Society (18).

The results of the gene expression analyses are summarized in Fig. 2: *GATA3* and *GATA6* are highly expressed in foetal and adult adrenal tissues, while *GATA4* is highly expressed in foetal and adult testis tissues. TARTs express high levels of *GATA3*, *GATA4* and *GATA6*, indicating adrenal- and testis-like expression patterns of GATA transcription factors.

### *GATA3* and *GATA6* mRNA levels can discriminate TARTs from LCTs, while protein levels cannot

**Figure 3:**
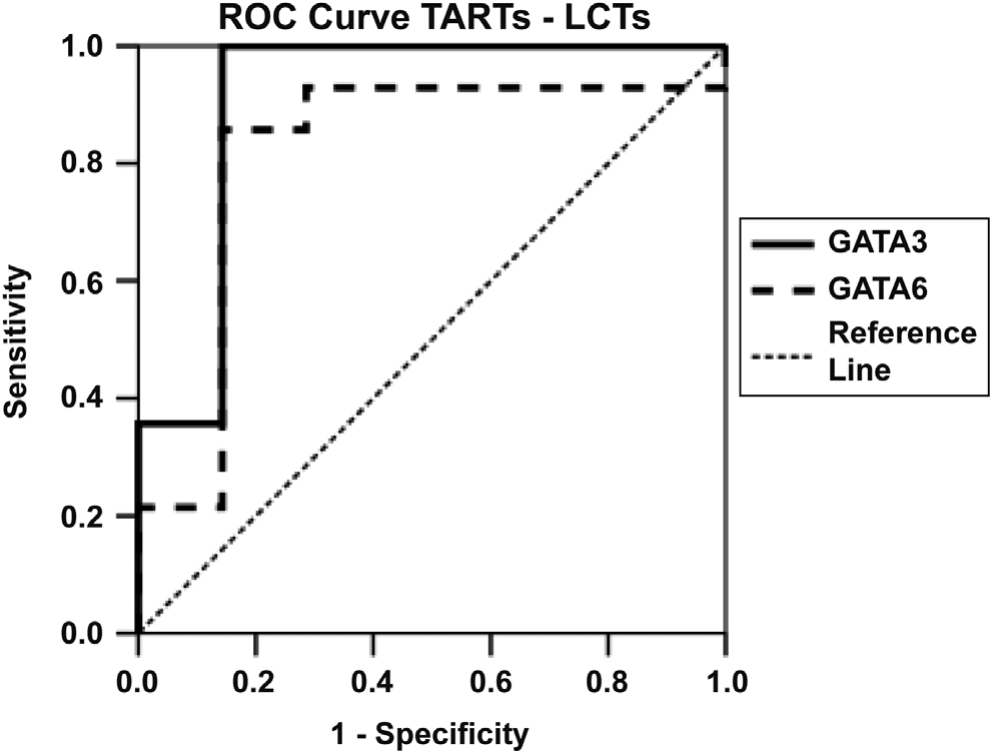
Discriminative potential of *GATA3* and *GATA6* gene expressions in distinguishing TARTs from LCTs. Receiver operating characteristic analyses were performed. An area under the curve of 0.908 was observed for *GATA3*, while this was 0.816 for *GATA6*. LCTs, Leydig cell tumours; TARTs, testicular adrenal rest tumours.

Next, we compared GATA expression and their discriminative potential between TART and LCT tissues, as there is currently no single marker that can distinguish between these two pathologies. *GATA3* gene expression was 15.8-fold higher expressed in TART compared to LCT tissues (*P* ≤ 0.01 Fig. 1B), and *GATA6* was 16.5-fold higher expressed in TARTs compared to LCTs (*P* ≤ 0.05 Fig. 1D), while *GATA4* showed no significant difference between TARTs and LCTs (Fig. 1C). To determine the discriminative potential in distinguishing TARTs from LCTs based on GATA3 or *GATA6* gene expression, we performed ROC analyses. *GATA3* showed excellent discriminative potential to differentiate TARTs from LCTs with an AUC of 0.908, while *GATA6* showed good discriminative potential with an AUC of 0.816 (Fig. 3).

**Figure 4:**
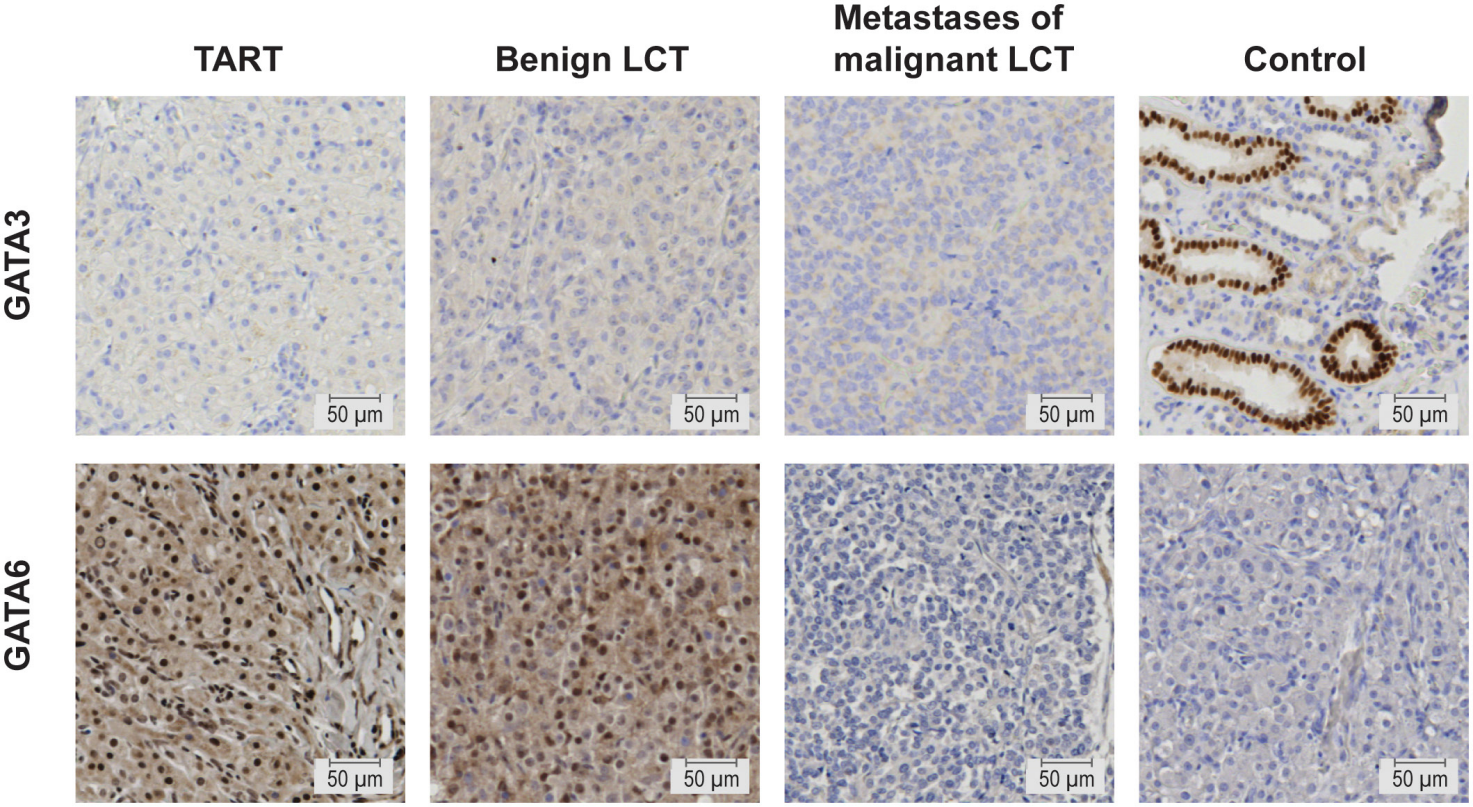
GATA3 and GATA6 protein expressions in human TARTs, benign LCTs, metastases of malignant LCTs and control tissues. GATA3 and GATA6 expressions (positive staining) are indicated by brown nuclei and/or brown cytoplasm. Protein expression of GATA3 is present in the positive control tissue (kidney), but absent in TARTs, benign LCTs and metastases of malignant LCTs. Protein expression of GATA6 is present in the cytoplasm and nuclei of TARTs and benign LCTs, however absent in metastases of malignant LCTs and the negative control (tissue slides without primary antibody). Representative pictures are shown. Scale bar represents a distance of 50 µm. LCTs, Leydig cell tumours; TARTs, testicular adrenal rest tumours.

To enhance the clinical applicability of GATAs as TART biomarkers and to determine which cells express GATA, we set out to assess GATA3 and GATA6 expressions using immunohistochemistry on paraffin-embedded formalin-fixed tissues. Protein expression was analysed in TARTs (*n* = 16), benign LCTs (*n* = 4) and metastases of malignant LCTs (*n* = 3). GATA3 protein expression was undetectable in all TART samples as well as in all LCTs, while the tubular cells of kidney sections (positive control) showed nuclear expression (Fig. 4). In TARTs and LCTs, GATA6 protein expression was heterogeneous (Supplementary Figs 1 and 2), while expression was absent in negative control samples (Fig. 4). Both nuclear and cytoplasmic stainings were observed, with a high variability in intensity and percentage of positive cells. TARTs and benign LCTs show similar intensity and percentage of cells with GATA6 protein expression, while protein expression of GATA6 in metastases of malignant LCTs is almost absent as there are only very few cells with staining (Fig. 4).

### GATA transcription factors and their possible role in the aetiology of TARTs

We hypothesized that prenatal exposure of foetal steroidogenic pluripotent cells to ACTH might induce TARTs via GATA transcription factors. ACTH acts on the ACTH receptor (MC2R), a G-coupled protein, using cAMP as a second messenger. Indeed, one or multiple CREB-binding sites occur inside the gene body or up to 10 kb upstream of the transcription start site of multiple *GATA* genes (http://sabiosciences.com/chipqpcrsearch.php?app=TFBS), suggesting that cAMP could be a GATA expression-inducing second messenger, involved in de- and/or upregulation of GATAs in TARTs.

**Figure 5:**
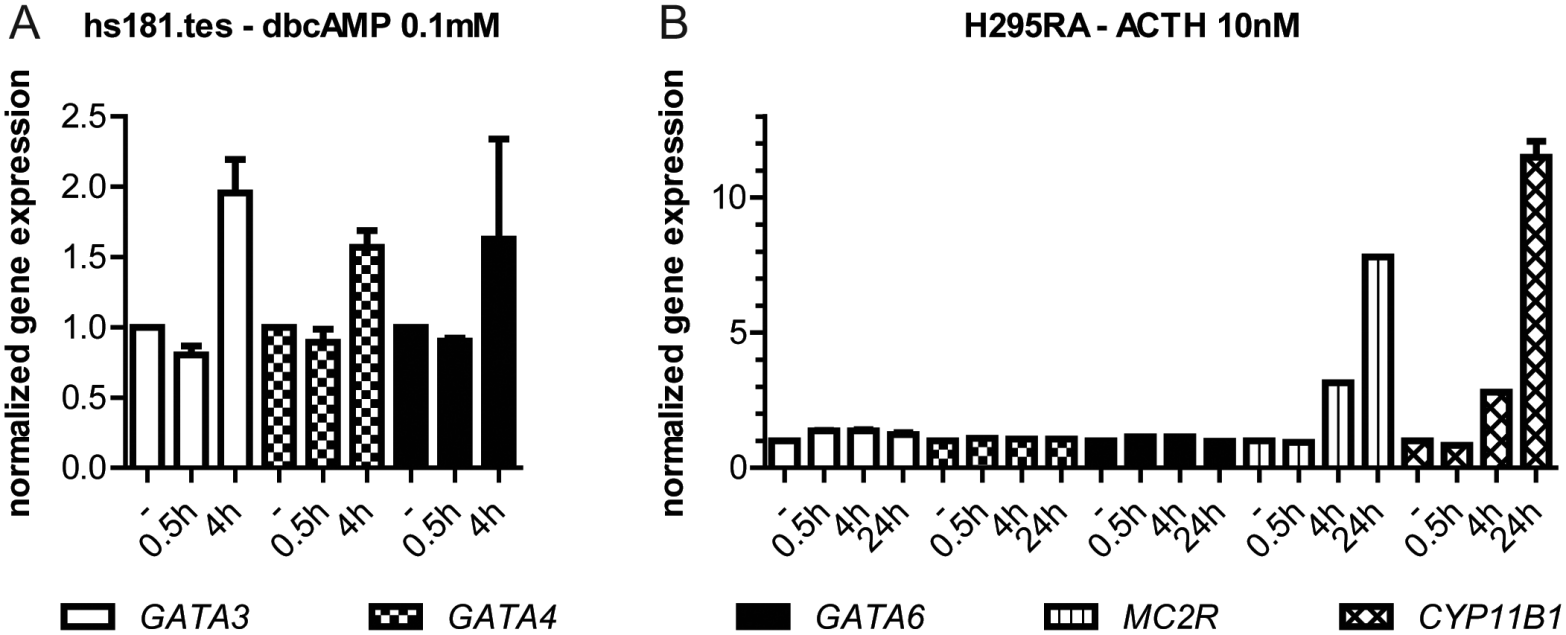
GATA transcription factors and their possible role in the aetiology of TARTs. Long-term exposure to elevated levels of ACTH is present in patients with congenital adrenal hyperplasia and this is associated with the development of TARTs. ACTH binds to its receptor (MC2R), using cAMP as a second messenger. CREB-binding sites are present within the gene body or up to 10 kb upstream of the transcription start site in GATA3, GATA4 and GATA6. cAMP could therefore be a GATA expression-inducing second messenger. Source: http://sabiosciences.com/chipqpcrsearch.php?app=TFBS. (A) Hs181.tes cells were incubated with 0.1 mM dibutyryl cAMP (Hs181.tes cells do not express ACTH receptor) for 0 min, 30 min or 4 h. Gene expression was calculated using the delta Ct method and corresponding *HPRT* expression was used to normalize. (B) H295RA cells were incubated with 10 nM ACTH for 0 min, 30 min, 4 h or 24 h. Delta Ct method and corresponding *HPRT* value were used to calculate normalized gene expression.

Incubation of a foetal testis cell line (hs181.tes) with 0.1 mM of dbcAMP for 4 h showed a moderate increase in gene expressions of *GATA3*, *GATA4* and *GATA6*, although this did not reach statistical significance (Fig. 5A). However, there is no *in vitro* model of foetal testis cells expressing MC2R. Therefore, we used an ACTH-sensitive adrenocortical cell line (H295RA).

ACTH incubation with 2 nM (results not shown) or 10 nM (Fig. 5B) for 30 min, 4 h or 24 h increased *MC2R* and *CYP11B1* gene expressions (positive controls), indicating that the system is indeed responsive to ACTH. However, no altered gene expressions for *GATA3*, *GATA4* or *GATA6* were found.

## Discussion

To the best of our knowledge, this is the first description of GATA transcription factors in human TARTs. TARTs expressed both testicular (*GATA4)* and adrenal (*GATA3* and *GATA6*) characteristics, thereby confirming our previous findings of both adrenal and testicular features of TARTs (6). Furthermore, differences in *GATA3* and *GATA6* mRNA expression levels might be used to discriminate TARTs (high) from LCTs (low expression), indicated by good AUCs (>0.8) in ROC analyses, although at the protein expression level, immunohistochemistry did not discriminate. In addition, as long-term exposure to elevated ACTH levels is linked to occurrence of TART (25, 26, 27), we hypothesized that (prenatal) exposure of (primordial) steroidogenic cells in the testes to ACTH might induce TARTs via deregulation of GATA transcription factors. Human foetal testis cells indeed show increased GATA expression after incubation with cAMP. However, adrenocortical cells (the only human ACTH-sensitive model available) did not show increased expression after ACTH incubation.

Although interesting to again find both adrenal- and testis-like characteristics of TARTs, this expression pattern does not correspond to the expression pattern observed in the adrenal-like cells of the GATA4/GATA6 double-knockout mice described earlier (19). The steroidogenic cells in the double-knockout mice should not express GATA4 and GATA6, as the model eliminated expression in all steroidogenic cells. In addition, the adrenal-like cells in these mice lacked *HSD17B3* and *INSL3* expressions, whereas these genes are expressed in human TARTs (19). Therefore, the observed gene expression patterns of adrenal-like cells found in these mice do not resemble the observed gene expression pattern of human TART.

We assessed the potential of GATA transcription factors as differential diagnostic tools to discriminate TARTs from LCTs, which are difficult to distinguish due to their morphological resemblance. Misdiagnosis can have profound consequences for the treatment of a patient with a testicular tumour (8, 11). Therefore, a clinical need for differential diagnostic markers that can differentiate between both pathologies exists. Bilateralism of the tumours (25, 30), presence of Reinke crystals (1, 5, 25, 31, 31, 32, 33, 34) and expressions of synaptophysin, Inhibin α, CD56, androgen receptor, DLK1, INSL3, CYP11B1, CYP21A2 and MC2R (33, 34, 35, 36) have all been studied as potential markers, but none of these markers individually can reliably discriminate TARTs from LCTs. We found significantly higher gene expressions of *GATA3* and *GATA6* in TARTs compared to LCTs with good discriminative potential. This suggests that measurement of these genes may be used in a diagnostic setting as a discriminative marker between TART and LCT tissues. To improve the usefulness of these markers in the clinic and to determine which cells express GATA, we determined protein expressions of GATA3 and GATA6 on paraffin-embedded tumour samples using standard immunohistochemical techniques. GATA3 protein expression, however, was undetectable in TARTs and LCTs. On the other hand, GATA6 protein expression was heterogeneous both within and between TART, benign LCT and metastases of malignant LCT tissue samples. We observed high variability in the location, intensity of staining and percentage of GATA6-positive cells. We identified very low expression of GATA6 in metastases of malignant LCTs, which might reflect a change in the status in the primary tumour cells promoting invasion. Nevertheless, GATA6 expression is heterogeneously expressed within and between TARTs and benign LCTs, and it can therefore not be used as a discriminatory biomarker for these pathologies.

We also determined *GATA* expression in human foetal adrenal and testis tissues. *GATA1* was the only GATA gene in which a significant change in expression was identified between foetal and adult adrenal tissues. We found *GATA4* expression in foetal and adult testis tissues, and *GATA6* expression in foetal and adult adrenal. Previous findings are in agreement with our data. Viger and coworkers summarized all known literature on GATA expression in adrenogonadal development, mainly based on mice models (18). However, as we studied human TARTs, we will focus on reported expression in human tissues. Ketola and coworkers found GATA4 and GATA6 mRNAs and protein expressions in human foetal testis tissues, although a decreasing trend with advanced foetal development was observed for GATA6 expression (37). In foetal testis tissues, *GATA4* was the predominantly expressed GATA gene. Jiminez and coworkers found *GATA6* expression in human adult adrenal tissues, but no *GATA4* expression (38). This is in agreement with Kiiverii and coworkers who showed *GATA4* and *GATA6* expressions in human foetal adrenal, but only GATA6 mRNA and protein expression in human adult adrenal (39). In summary, and in correspondence with known literature, we found expression of *GATA4* in foetal and adult testis tissues, and *GATA6* expression in foetal and adult adrenal tissues. In addition, we found *GATA3* expression in foetal and adult adrenal tissues.

We found high relative gene expression levels of *GATA3*, *GATA4* and *GATA6* in TARTs, suggesting that dysregulation of these transcription factors is involved in the aetiology of TARTs. GATA genes contain one or several CREB sites and previously cAMP was described to induce GATA4 and GATA6 expressions in the gonadal cell lines MSC-1, mLT and MA-10 (22, 23, 24). We therefore stimulated foetal testis cells with cAMP and indeed found moderate increase in expressions of *GATA3*, *4* and *6*. The ACTH receptor (MC2R) signals via cAMP and elevated ACTH levels are present in CAH patients and associated with the development of TARTs (25, 26, 27). We hypothesized that dysregulation of GATA expression by ACTH in foetal steroidogenic cells could eventually lead to the formation of TARTs. Regretfully, no human foetal cell lines are available that express functional MC2R. Human cell lines, in general, tend to have low (functional) ACTH receptor expression, although several adrenocortical cell lines, including H295R cells, are known to have at least moderate expression of MC2R (40, 41). ACTH responsiveness in H295R cells was increased by over-expression of MRAP, resulting in the H295RA cell line, the only human ACTH-sensitive cell line available (28). We are aware of the limitation of using a differentiated cell line as a model for the involvement of ACTH in foetal steroidogenic cells. In the present study, we demonstrated that ACTH does not influence *GATA* expression in H295RA cells. Timing of ACTH exposure might play an important role in GATA dysregulation. Therefore, dysregulation of GATA might still be a key player in the formation of TART and is possibly regulated by ACTH prenatally.

In conclusion, testis-like expression of *GATA4* and adrenal-like expressions of *GATA3* and *GATA6* were observed in TARTs, suggesting that dysregulation of GATA transcription factors in a pluripotent foetal cell is involved in TART formation. Furthermore, gene expression of GATA transcription factors showed good discriminative potential to differentiate TARTs from LCTs, but further studies have to be performed establishing thresholds in less-invasive material such as blood or urine to be of applicable use.

## Supplementary Material

Supporting Figure 1

Supporting Figure 2

Supporting Table 1

## Declaration of interest

The authors declare that there is no conflict of interest that could be perceived as prejudicing the impartiality of the research reported.

## Funding

This work was supported by the International Fund Congenital Adrenal Hyperplasia (2016 IFCAH grant).
