# Supplementary figures and images for: GATA transcription factors in testicular adrenal rest tumours
Source: Endocr Connect. 2017 Oct 16;6(8):866–75. doi: 10.1530/EC-17-0215 (PMC5682415; doi:10.1530/EC-17-0215)

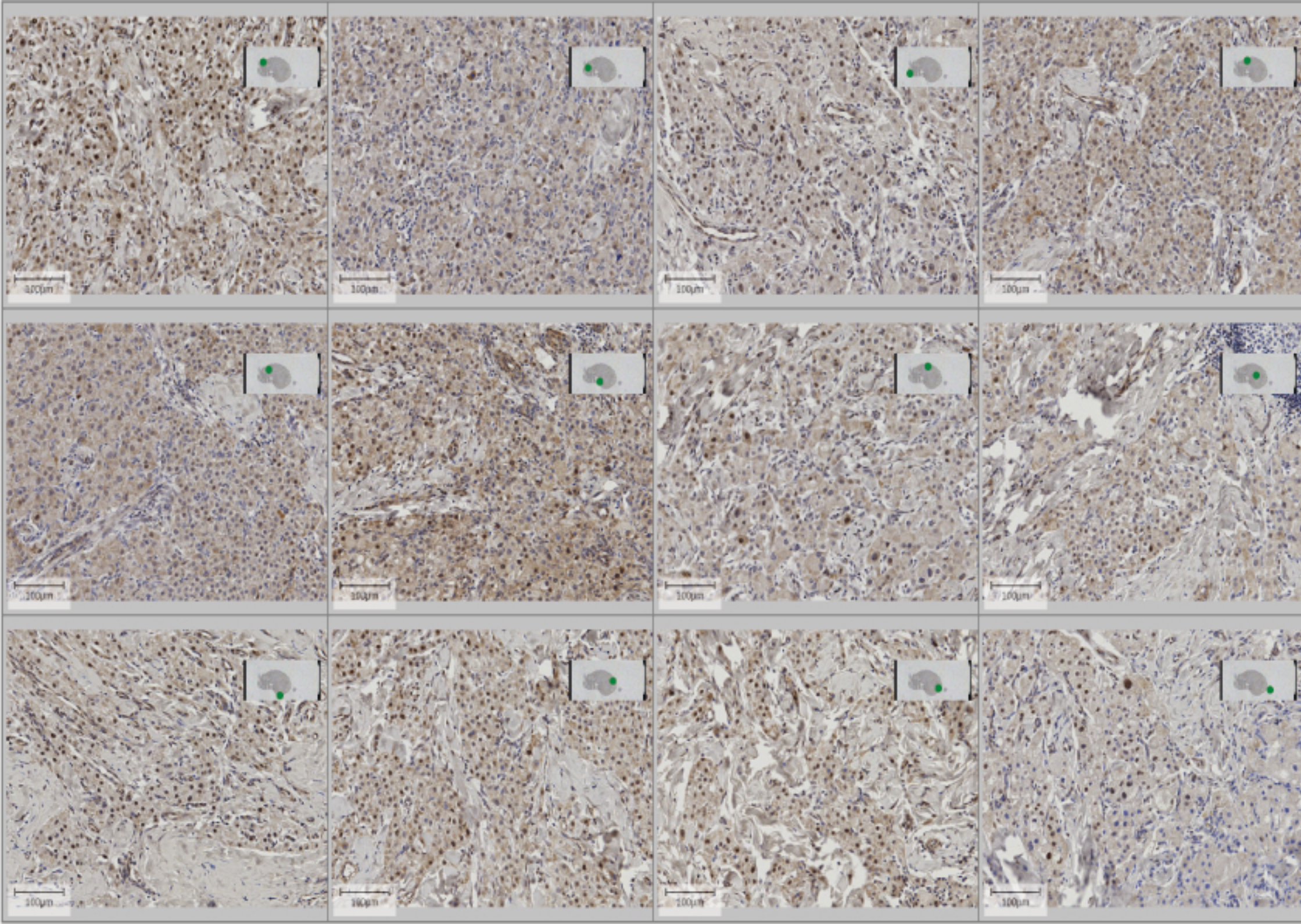

Supplement: Supporting Figure 1 [file ec-6-866-s001.pdf]

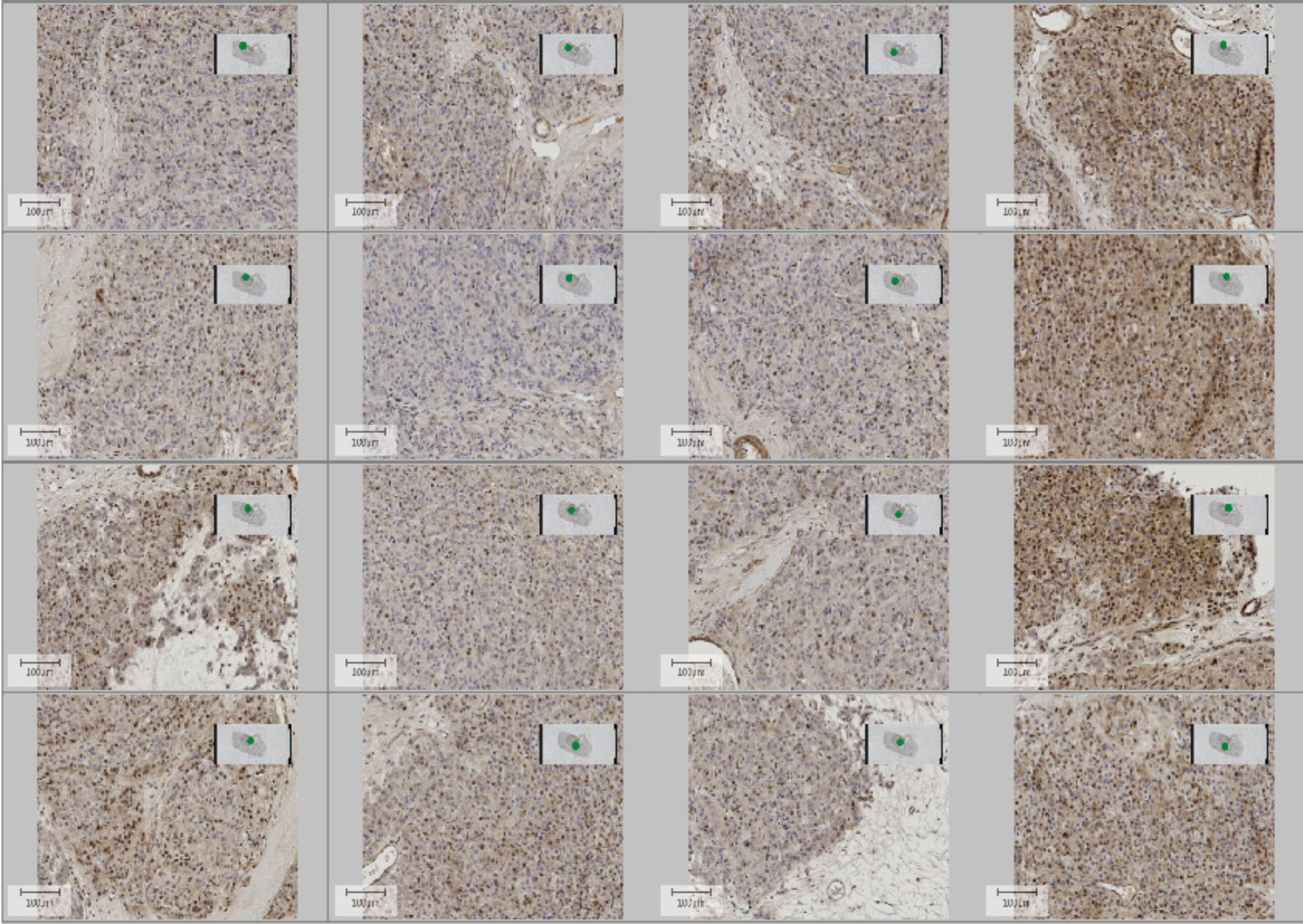

Supplement: Supporting Figure 2 [file ec-6-866-s002.pdf]
