# Supplementary material for: GATA transcription factors in testicular adrenal rest tumours
Source: Endocr Connect. 2017 Oct 16;6(8):866–75. doi: 10.1530/EC-17-0215 (PMC5682415; doi:10.1530/EC-17-0215)
Supplement: Supporting Table 1 [file ec-6-866-t001.pdf]

Supplemental Table S1: Primer sequences and specifications.

Genes were searched in the NCBI database and imported in PerlPrimer [Marshall2004]. Primers were derived based on an amplicon size of 75-125 base pairs, a length of 17-24 nucleotides, a GC-content of 40-65%, melting temperature of 56-62 °C, an exon-exon junction spanning amplicon and where possible boundary-overlapping primers. Primers were checked for dimers, hairpins and secondary amplicon structures and a set of forward and reverse primers for each gene was ordered (Biolegio, Nijmegen, the Netherlands). The *HPRT* gene was used as normalizing gene [de Kok2005]. Sequences and specifications of all primers are listed in Supplemental Table 1. Efficiency and specificity of the primers were tested using the IQ™SYBR® Green Supermix on a CFX96 Touch Real-Time PCR Detection System (Bio-Rad Laboratories, Veenendaal, Netherlands). The PCR products were checked for quality and product size by melting curve analysis and agarose gel electrophoresis.

Gene	Accession Number	Sequence 5'→ 3'	Exon boundary overlapping
GATA1<br>*GATA binding protein 1 (globin transcription factor 1)*	NM_002049.3	FP: AAGAAGCGCCTGATTGTC<br>RP: GCATTTCTCCGCCACAG	yes
GATA3<br>*GATA binding protein 3*	NM_001002295.1<br>NM_002051.2	FP: CAGACCACCACAACCACAC<br>RP: TGCCTTCCTTCTTCATAGTCAG	no
GATA4<br>*GATA binding protein 4*	NM_001308093.1<br>NM_002052.4	FP: TCTACATGAAGCTCCACGGG<br>RP: TATTCAGGTTCTTGGGCTTCC	no

	NM_001308094.1		
GATA6<br>*GATA binding protein 6*	NM_005257.5	FP: GAGGGAATTCAAACCAGGA<br>RP: GTTGGAGTCATGGGAATGG	no
MC2R<br>*melanocortin 2 receptor*<br>*(ACTH receptor)*	NM_000529.2	FP:<br>CAGAGCTGAAGGTGATTGGGA<br>RP: AAGGCGGGGATGTTACTTGG	no
CYP11B1<br>*11β-hydroxylase*	NM_000497.3	FP: GGCAGAGGCAGAGATGCTG<br>RP:<br>TCTTGGGTTAGTGTCTCCACCTG	yes
HPRT	NM_000194.2	FP:<br>TATTGTAATGACCAGTCAACAG<br>RP: GGTCCTTTTCACCAGCAAG	yes
